# *Reynoutria japonica* Houtt for Acute Respiratory Tract Infections in Adults and Children: A Systematic Review

**DOI:** 10.3389/fphar.2022.787032

**Published:** 2022-02-24

**Authors:** Zhi-Jie Wang, Jeanne Trill, Lin-Lin Tan, Wen-Jing Chang, Yu Zhang, Merlin Willcox, Ru-Yu Xia, Yue Jiang, Michael Moore, Jian-Ping Liu, Xiao-Yang Hu

**Affiliations:** ^1^ Department of Oncology, Shanxi Province Hospital of Tradition Chinese Medicine, Taiyuan, China; ^2^ The Institute of Shanxi Traditional Chinese Medicine, Taiyuan, China; ^3^ Primary Care Research Centre, Faculty of Medicine, University of Southampton, Southampton, United Kingdom; ^4^ Center for Evidence-based Chinese Medicine, Beijing University of Chinese Medicine, Beijing, China; ^5^ The First Affiliated Hospital of Anhui University of Chinese Medicine, Hefei, China

**Keywords:** *reynoutria japonica*, herbal remedy, respiratory tract infections (RTIs), randomized controlled trials, meta-analysis

## Abstract

**Introduction:** Respiratory tract infections (RTIs) are a major cause of morbidity and mortality in some high-risk groups including children and older adults. There is evidence that Chinese herbal medicine has an effect on RTIs. *Reynoutria japonica* Houtt (better known under its synonym *Fallopia japonica* (Houtt.) Ronse Decr.) (*F. japonica*), a commonly used Chinese herbal medicine, has a high content of resveratrol and glycosides. In traditional Chinese medicine theory, *F. japonica* has the effect of clearing heat in the body, improving blood and qi circulation, eliminating phlegm, and relieving cough, so it may have an effect on RTIs.

**Methods:** This systematic review was registered under PROSPERO CRD42020188604. Databases were searched for randomized controlled trials of *F. japonica* as a single herb, or as a component of a complex herbal formula for RTIs. Quality of methodology was assessed by two reviewers independently using the Cochrane Risk of Bias Tool. The primary outcome was symptom improvement rate. The secondary outcome measures were fever clearance time, Murray lung injury score and incidence of adverse effects. The extracted data were pooled and meta-analysed by RevMan 5.3 software.

**Results:** Eight RCTs with 1,123 participants with acute RTIs were included in this systematic review, and all the RCTs used *F. japonica* as part of a herbal mixture. Only one included trial used *F. japonica* in a herbal mixture without antibiotics in the treatment group. The findings showed that herbal remedies that included *F. japonica* could increase the symptom improvement rate (risk ratio 1.14, 95% confidence intervals [1.09, 1.20], I^2^ = 0%, *p* < 0.00001, n = 7 trials, 1,013 participants), shorten fever duration, reduce Murray lung injury score and did not increase adverse events (RR 0.33, 95% CI [0.11, 1.00], I^2^ = 0%, *p* = 0.05, n = 5 trials, 676 participants).

**Conclusion:** There is limited but some evidence that *F. japonica* as part of a herbal mixture may be an effective and safe intervention for acute RTIs in clinical practice. In future studies it would be preferable to evaluate the effectiveness and safety of using *F. japonica* without antibiotics for acute RTIs.

## 1 Introduction

A variety of viruses and bacteria can cause respiratory tract infections (RTIs) including upper and lower RTIs. The most frequent upper RTIs are the common cold, laryngitis, tonsillopharyngitis, and otitis media; lower RTIs include bronchitis, bronchiolitis, and pneumonia. RTIs are a major cause of morbidity and mortality in some high-risk groups including children and older adults (Shek and Lee, 2003). RTIs are the leading infectious cause of death, and the sixth-leading cause of death overall worldwide. RTIs result in millions of clinical visits and subsequent prescriptions of antibiotics every year. Globally, in 2016 there were more than 300 million cases of RTIs (GBD 2016 Lower Respiratory Infections Collaborators, 2018).

Symptom relief is often the target of treatment for RTIs. Appropriate and effective treatments may limit cough, fever, pain, congestion and other symptoms in patients with RTIs (Irwin et al., 2006). Rates of prescribing antibiotics for respiratory conditions in the United Kingdom are high with median prescribing rates of 54% (Gulliford et al., 2014). Of the antibiotics prescribed in primary care with an attributable target, nearly half were for respiratory conditions (Costelloe et al., 2010) with similar high rates reported in China (Li, 2019), despite the lack of evidence to support using antibiotics for viral infections (Weintraub, 2015). The overuse of antibiotics increases the risk of colonisation with resistant bacteria, promotes antibiotic resistance in the community, risks subsequent infection with antibiotic resistant organisms, and may cause some allergic reactions and other adverse effects (Weintraub, 2015; Bryce et al., 2016).

Chinese herbal medicine (CHM) is an important part of Traditional Chinese Medicine (TCM), and is used for almost all kinds of diseases in clinical practice in China. CHMs include one or more herbs for syndromes or disorders according to the TCM theories (Wu et al., 2008). The Food and Drug Administration (FDA) in the United States has approved the use of 13 herbal remedies, and of the total 252 drugs in the World Health Organization (WHO) essential medicine list, 11% are exclusively of plant origin, resulting in increased sales of CHMs (Wachtel-Galor and Benzie, 2011). Considering the adverse effects and resistance of antibiotics, and the diverse symptoms of RTIs, CHMs are commonly used for inflammation including RTIs (Chen et al., 2006).

Hu Zhang [*虎杖*] in the *Chinese Pharmacopoeia* consists of the roots and rhizomes of Japanese Knotweed. The accepted scientific name of this plant is now *Reynoutria japonica* Houtt, but it also has many synonyms, of which the most important are *Fallopia japonica* (Houtt) Ronse Decr. and *Polygonum cuspidatum Siebold & Zucc*. The herb is commonly used in CHM therapy (Jeong et al., 2010), and dates back to at least the Han Dynasty when it was recorded in the ‘Supplementary Records of Famous Physicians’ [*名医别录*]. The herb is used for clearing heat from the body, improving blood [*血*] and qi [*气*] circulation, eliminating phlegm, and relieving cough and asthma (Yan et al., 2019). It is always classed as a sovereign herb (a herb that plays a major role in the treatment of the main syndrome or main symptom) in CHM formulas for treating RTIs, an example of which is Shufeng Jiedu capsule. It is used for nervous system disorders, bronchitis, high blood pressure and jaundice (Editor Committee of Jiangsu New Medical College, 2011.).

*F. japonica* is reported to have antiallergic, antimutagenic, antioxidant, antibacterial and antiviral activities, and the main active compounds are believed to be resveratrol and glycosides (Bralley et al., 2008; Zahedi et al., 2013; Goc et al., 2015). Some activation of nuclear transcription factors such as Nuclear Factor kappa B (NF-κB), activator protein-1, matrix metalloproteinase-9 (MMP-9) and tissue inhibitor of metalloprotease-1 (TIMP-1) were reported in connection with RTIs, which might be activated by *F. japonica* (Manna et al., 2000; Huang, 2017). Polydatin (PD) is another major active ingredient of *F. japonica*; the herb is widely used for treating both acute and chronic lung disorders (Lin et al., 2011; Lee et al., 2015).

*F. japonica* and its extracts have been reported to have a positive effect on RTIs, but no systematic review has ever been conducted on its use for these conditions. In this review, we aimed to evaluate the effectiveness and safety of *F. japonica,* or herbal remedies that included *F. japonica,* for acute RTIs in adults and children.

## 2 Method

The Preferred Reporting Items for Systematic Reviews and Meta-Analyses (PRISMA) has been adhered to in reporting this review (Moher et al., 2015), and the protocol has been registered under PROSPERO (CRD42020188604).

### 2.1 Data Sources and Search Terms

A search was carried out across the databases MEDLINE, embase, Cochrane Central Register of Controlled Trials, Allied and Complementary Medicine Database (AMED), Web of Science, CINAHL Plus, China National Knowledge Infrastructure (CNKI), Wan Fang, Chinese Science and Technology Journal Database (VIP), Sino-Med Database Research Information Service System (RISS), Oriental Medicine Advanced Searching Integrated System (OASIS), and the National Assembly Library from their inception to July 2021. Clinical trial registers, ClinicalTrials.gov and the World Health Organization International Clinical Trials Registry Platform, were also searched. Search terms included ‘*Fallopia japonica’* or ‘Hu Zhang’ or ‘Japanese Knotweed’ or ‘*Reynoutria japonica’* or ‘*Polygonum cuspidatum’*, AND ‘respiratory tract infections’ or ‘common cold’ or ‘cough’. Additional search terms and strategies in different languages with different databases are listed in Supplementary Appendix S1. We also repeated the searches including the names of the complex formulae which included *F. japonica*.

### 2.2 Study Selection

#### 2.2.1 Inclusion and Exclusion Criteria

This systematic review included published and unpublished randomized controlled trials (RCTs), and data from crossover trials prior to the crossover. Controlled before and after studies, interrupted time series studies, quasi-RCTs and non-experimental studies were not included due to their potential high risk of bias.

Population: Trials with patients in all age groups, with either an acute respiratory tract infection (ARI) diagnosis or presentation with ARI symptoms were included. A clinical diagnosis of ARI was the main inclusion criterion. Diagnoses of upper or lower ARIs included the common cold, otitis media, influenza, rhinosinusitis, laryngitis, tonsillitis, pharyngitis, supraglottitis, croup, tracheitis, bronchitis, and acute exacerbations of either asthma or chronic obstructive pulmonary disease (COPD). Symptoms of ARIs are defined as having symptoms such as cough, sore throat, fever, runny nose, earache and discoloured sputum with duration of less than 3 weeks. In and out patients were both included.

Trials were excluded if they recruited participants with another non infectious condition such as asthma or participants with infections such as tuberculosis and pneumonia which require antibiotics. Exclusion was applied to trials that included patients who had a known immune deficiency.

Intervention: any form of *F. japonica*, including oral, nasal, or external use, apart from injection; either as a single herb, or within a herbal remedy. There was no limitation concerning dosage, dosing method or duration of administration.

Comparator: no intervention, placebo or usual care such as antipyretics, antivirals, antibiotics, anti-inflammatories, steroids or corticosteroids were included.

#### 2.2.2 Outcome Measures

The primary outcome measure was effect estimation (symptom improvement rate). The secondary outcome measures were fever clearance time, lung injury score (such as the Murray lung injury score) and incidence of adverse effects.

#### 2.2.3 Data Selection and Collection

Literature searching and screening (titles, abstracts and full texts) was conducted by three reviewers independently (WZJ, XRY, JY), and disagreements were resolved through discussion and consensus, or were assessed by a fourth reviewer (HXY). There were no restrictions on language. Researchers were not blinded to the authors’ affiliations, journal of publication, or study results.

#### 2.2.4 Data Extraction and Management

Two reviewers (WZJ, ZY) independently extracted data from the included trials including study characteristics, participants and diseases, details of interventions on all trial arms, outcome measures, and adverse events.

### 2.3 Assessment of Bias and Reporting Quality of Included Trials

Two reviewers (WZJ, ZY) independently assessed the risk of bias using the Cochrane Collaboration risk of bias tool (Higgins et al., 2019). The risk of bias tool assessed seven domains and for each domain the two reviewers made a judgment whether the risk of bias was high, unclear or low. Disagreements were discussed and resolved with reference to the original protocol and, if necessary, arbitration by a third reviewer (HXY). We planned to conduct funnel plot tests for asymmetry to investigate potential reporting bias if this was feasible and there existed sufficient studies (≥10) under a single meta-analysis (Egger et al., 1997). The evidence level of the included trials was assessed by Grades of Recommendations Assessment, Development and Evaluation (GRADE) with the high, moderate, low or very low level.

### 2.4 Measures of Treatment Effect

Where possible, the analyses were based on intention to treat (ITT) data on each outcome provided for every randomized participant from the individual trials. For continuous outcomes, the end of treatment scores rather than change from baseline scores were extracted; for continuous data, due to the anticipated variability in the populations and interventions of included trials, a generic inverse variance random effects model was used to pool the mean differences (MD) with 95% confidence intervals (CI) to incorporate heterogeneity (Murad et al., 2015). If the units of the outcome measures used across studies were not consistent, the effects as standardized mean differences (SMD) were reported. An overall effect size of 0.2–0.5 was regarded as small, 0.5–0.8 as moderate and more than 0.8 as large. For dichotomous data, a random effects method was used to pool the summary risk ratio (RR) with 95% CI.

### 2.5 Dealing With Missing Data

Where standard deviation was not reported with means, it was calculated from the information reported such as CI, *p*-values, or F-values. ITT analysis was utilized for all outcomes as far as possible. For the missing data, we planned to contact the corresponding author of the original study.

### 2.6 Assessment of Heterogeneity

Between-study heterogeneity was assessed using the I^2^—statistic which describes the percentage of variation across studies due to heterogeneity rather than chance. Criteria recommended for interpretation of this statistic suggested that I^2^>30% represents moderate heterogeneity, I^2^>50% represents substantial heterogeneity and I^2^>75% represents considerable heterogeneity (Higgins et al., 2019). Where I^2^ values were above 50%, potential sources of heterogeneity were further investigated in a subgroup analysis. This was taken into account when interpreting the findings. As high levels of heterogeneity were to be expected due to complexity in the form of *F. japonica* (such as variation of the type of preparation and the percentage of the active ingredient), a random effects model was utilized to pool the overall effects (Higgins et al., 2019).

### 2.7 Sensitivity Analysis

Sensitivity analyses were performed for the primary outcome on usage of antibiotics, as well as on the overall RTI symptoms or two target symptoms: cough and sore throat. This was to determine whether the review conclusions would have differed if eligibility was restricted to trials with low risk of selection bias (Higgins et al., 2019).

### 2.8 Subgroup Analysis

Where sufficient data were available, several subgroup analyses were planned to compare the effect estimates between studies that evaluated: adults versus children (younger than 18); *F. japonica* in different preparations, e.g., granule versus capsule or other forms; *F. japonica* as a monotherapy versus as part of a complex herbal remedy, or a supplement mixture; or specific ARIs (e.g., tonsillitis, otitis media, rhinosinusitis, etc.) to be grouped together according to the main symptoms depending on the number of papers found.

## 3 Results

**FIGURE 1:**
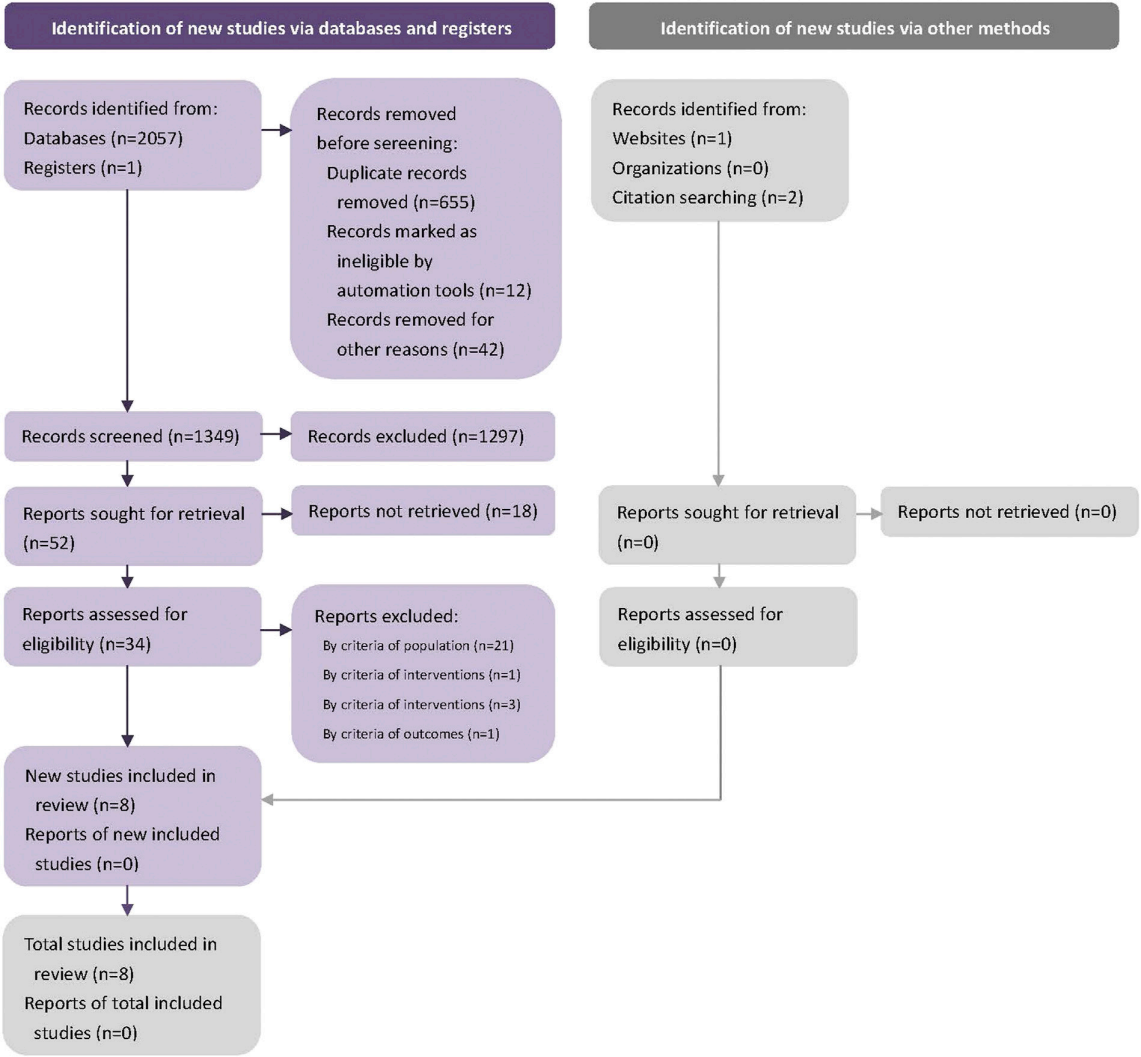
Flow diagram.

2061 potential studies were searched initially, and 712 duplicates were removed. The remaining 1,349 studies were screened by the title and abstract. 1,313 studies were excluded at this stage, 34 studies were screened in full text, and eight RCTs were included in the final systematic review (Li et al., 2014; Bi and Feng, 2015; Chen et al., 2016; Yang, 2019; Yang et al., 2019; Liu et al., 2020). (Figure 1). We contacted the corresponding author of the data missing studies, but no replies from the authors. Figure 1. Flow diagram.

### 3.1 Study Characteristics

Eight RCTs and 1,123 participants (which ranged between 84 and 237 in each trial) were included in this review (Table 1). All were carried out in China. No trial used *F. japonica* as a monotherapy, but all tested herbal remedies containing *F. japonica*. Three RCTs focused on children under 10 years old Li et al., 2014; Bi and Feng, 2015; Chen et al., 2016), and five focused on adults (Yang, 2019; Yang et al., 2019; Liu et al., 2020; Zhao and Wang, 2020; Zhang et al., 2021). Three trials (Bi and Feng, 2015; Chen et al., 2016; Yang et al., 2019) reported on treatment of inpatients, while others did not report whether patients were inpatients or outpatients. The treatment duration was from 3 to 45 days, and the most common was 3–7 days. Six trials tested Shufeng Jiedu capsule (Bi and Feng, 2015; Chen et al., 2016; Yang et al., 2019; Liu et al., 2020; Zhao and Wang, 2020; Zhang et al., 2021), one used Tuire liquid (Li et al., 2014), and one used Xuanfei Quyu Tongluo liquid (Yang, 2019). One trial (Zhang et al., 2021) used usual care plus Shufeng Jiedu capsule placebo, and other trials used usual care only in the control groups. Seven RCTs evaluated symptom improvement rate (Bi and Feng, 2015; Chen et al., 2016; Yang et al., 2019; Liu et al., 2020; Zhao and Wang, 2020; Zhang et al., 2021), three assessed the lung injury score (Yang, 2019; Yang et al., 2019; Liu et al., 2020), and three observed fever resolution time (Bi and Feng, 2015; Chen et al., 2016; Zhao and Wang, 2020). Five of the included trials reported adverse events, with two of them (Yang et al., 2019; Liu et al., 2020) reporting no adverse events. One (Bi and Feng, 2015) reported two cases (one nausea and one rash), one (Chen et al., 2016) reported one case of nausea, and one (Zhao and Wang, 2020) reported one case of diarrhoea following ingestion of Shufeng Jiedu capsule. None of the including trials reported the source of funding.

**TABLE 1:** Study characteristics.

study ID	Condition	setting	sample size	Mean age (±SD)		Gender (male/female)		Treatments		Outcome measures	Adverse events	source of funding
			T/C	T	C	T	C	T	C			
**URTIs**												
Bi and Feng, (2015)	pediatric URTIs	inpatient	84 (42/42)	5.5 ± 1.3 Y	6.0 ± 1.5 Y	24/18	22/20	UC + SFJDC for 3d	UC	symptom improvement rate, time without fever	2 (1 nausea, 1 rash)/8 (4 nausea, 2 diarrhea, 2 rash)	NR
Chen et al. (2016)	pediatric URTIs	inpatient	156 (78/78)	6.3 ± 3.5 Y	6.5 ± 1.5 Y	35/43	41/37	UC + SFJDC for 7d	UC	symptom improvement rate, time without fever	1 (nausea)/1 (rash)	NR
Li et al. (2014)	pediatric URTIs	unclear	100 (50/50)	4.91 ± 0.95 Y	4.77 ± 0.77 Y	23/27	24/26	Tuire Liquid for 6d	UC	symptom improvement rate	NR	NR
Liu et al. (2020)	acute URTIs	unclear	180 (90/90)	34.77 ± 7.24 Y	32.60 ± 8.95 Y	47/43	49/41	UC + SFJDC for 3d	UC	symptom improvement rate, lung function	No/No	NR
Zhao and Wang, (2020)	acute URTIs	unclear	156 (78/78)	37.30 ± 6.50 Y	36.80 ± 6.20 Y	41/37	40/38	UC + SFJDC for 5d	UC	symptom improvement rate, time without fever	1 (1 diarrhea)/3 (2 nausea, 1 fatigue)	NR
Zhang et al. (2021)	acute URTIs	unclear	237 (118/119)	31.94 ± 6.50 Y	35.94 ± 3.50 Y	67/51	65/54	UC + SFJDC for 3d	UC + SFJDC placebo	symptom improvement rate	NR	NR
**AECOPD**												
Yang (2019)	AECOPD	inpatient	100 (50/50)	61.30 ± 4.70 Y	63.15 ± 3.71 Y	27/23	24/26	UC + SFJDC for 7d	UC	symptom improvement rate, lung function	No/No	NR
Yang, (2019)	AECOPD	unclear	110 (55/55)	71.23 ± 2.13 Y	71.30 ± 1.98 Y	30/25	31/24	UC + Xuanfei Quyu Tongluo Liquid for 45d	UC	lung function	NR	NR

SFJDC: Fallopia japonica root [虎杖], Forsythia suspensa fruit [连翘], Isatis indigotica L root [板蓝根], Bupleurum chinense root [柴胡], Patrinia scabiosaefolia flower [败酱草], Verbena officinalis L. herb [马鞭草], Phragmites communis rhizome [芦根], Glycyrrhiza uralensis Fisch root [甘草]. Tuire Liquid: Indigo naturalis [青黛], Herba Menthae [薄荷], Armeniaca amarum seed [杏仁], Fallopia japonica root [虎杖], Artemisia annua herb [青蒿], Crystalline Mirabilite [寒水石], Forsythia suspensa fruit [连翘], Uncaria spp. twigs with hooks [钩藤], Cassia occidentalis seed [望江南], Fortunes Boss Fern Rhizome [贯众], Fructus Crataegi [山楂], Massa Medicata Fermentata [神曲]. Xuanfei Quyu Tongluo Liquid: Salvia miltiorrhizae root [丹参], Deer antler glue [鹿角胶], Armeniaca amarum seed [杏仁], Pinellia ternata (Thunb.) Breit root [半夏], Wolfiporia extensa (Peck) Ginns fungus[茯苓], Trichosanthes spp. fruit[瓜蒌], Psoralea corylifolia Linn fruit [补骨脂], Fallopia japonica root [虎杖], Ephedra [麻黄], Citrus reticulata Blanco fruit peel [陈皮]. The formula of the included herbal remedies. T: treatment group; C: control group; UC: usual care; NR: not reported; URTIs: upper respiratory tract infections; AECOPD: acute exacerbation of chronic obstructive pulmonary disease; SFJDC: shufeng jiedu capsule.

### 3.2 Risk of Bias

**FIGURE 2:**
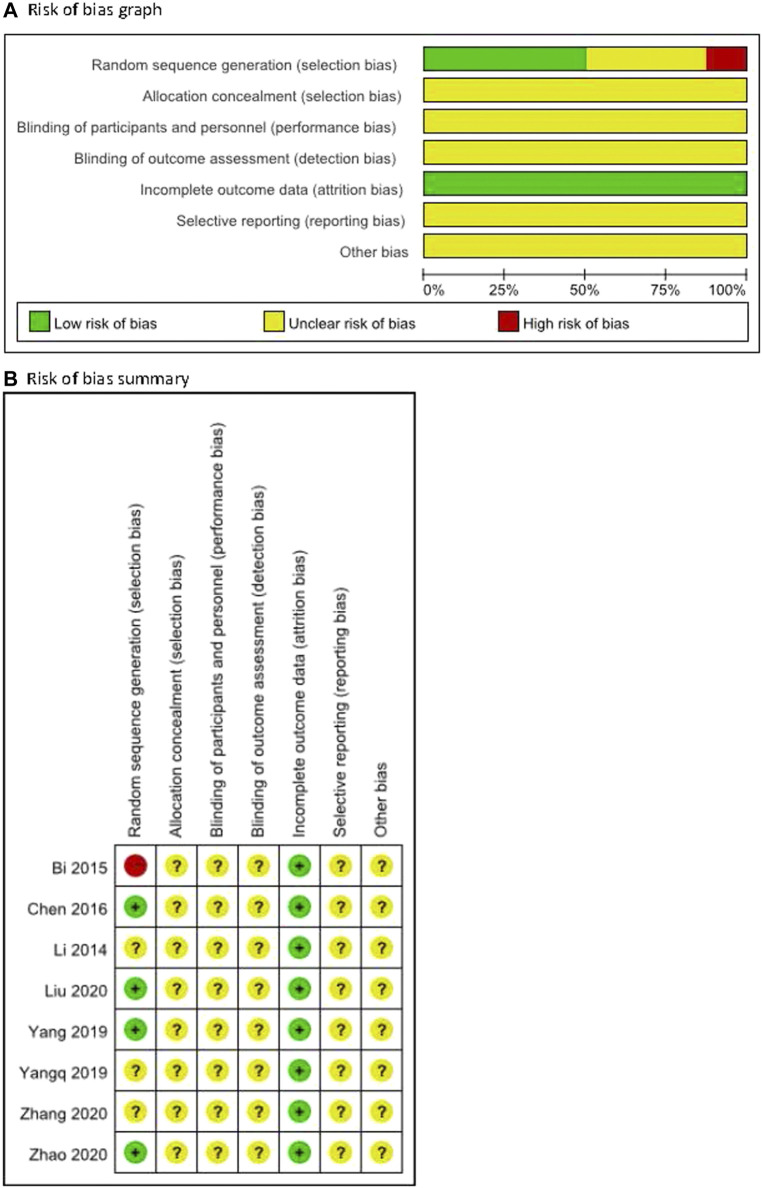
Risk of bias of the including trials.

The methodological quality for all included trials was poor (Figure 2). For random sequence generation, four trials were judged low risk bias as random number tables or SAS software was utilized (Chen et al., 2016; Yang et al., 2019; Liu et al., 2020; Zhao and Wang, 2020). One was judged to be at high risk of bias because of an inadequate method (the sequence in relation to seeing a doctor) for random sequence generation (Bi and Feng, 2015), while others were not considered to use clear methods. For allocation concealment, the risk of all included trials was unclear because they did not report the information. For blinding, one trial (Zhang et al., 2021) reported double blinding without detailed information so the risk was judged as unclear, and others did not report the information on blinding of outcome assessment so the risk was judged as unclear for all. All trials reported full information on outcome data, but there was incomplete information on selective reporting and other potential biases such as criteria for disease or participants, ethics for conducting a clinical trial, funding, or conflict of interest.

### 3.3 Effect Estimation

#### 3.3.1 Symptom Improvement Rate

**FIGURE 3:**
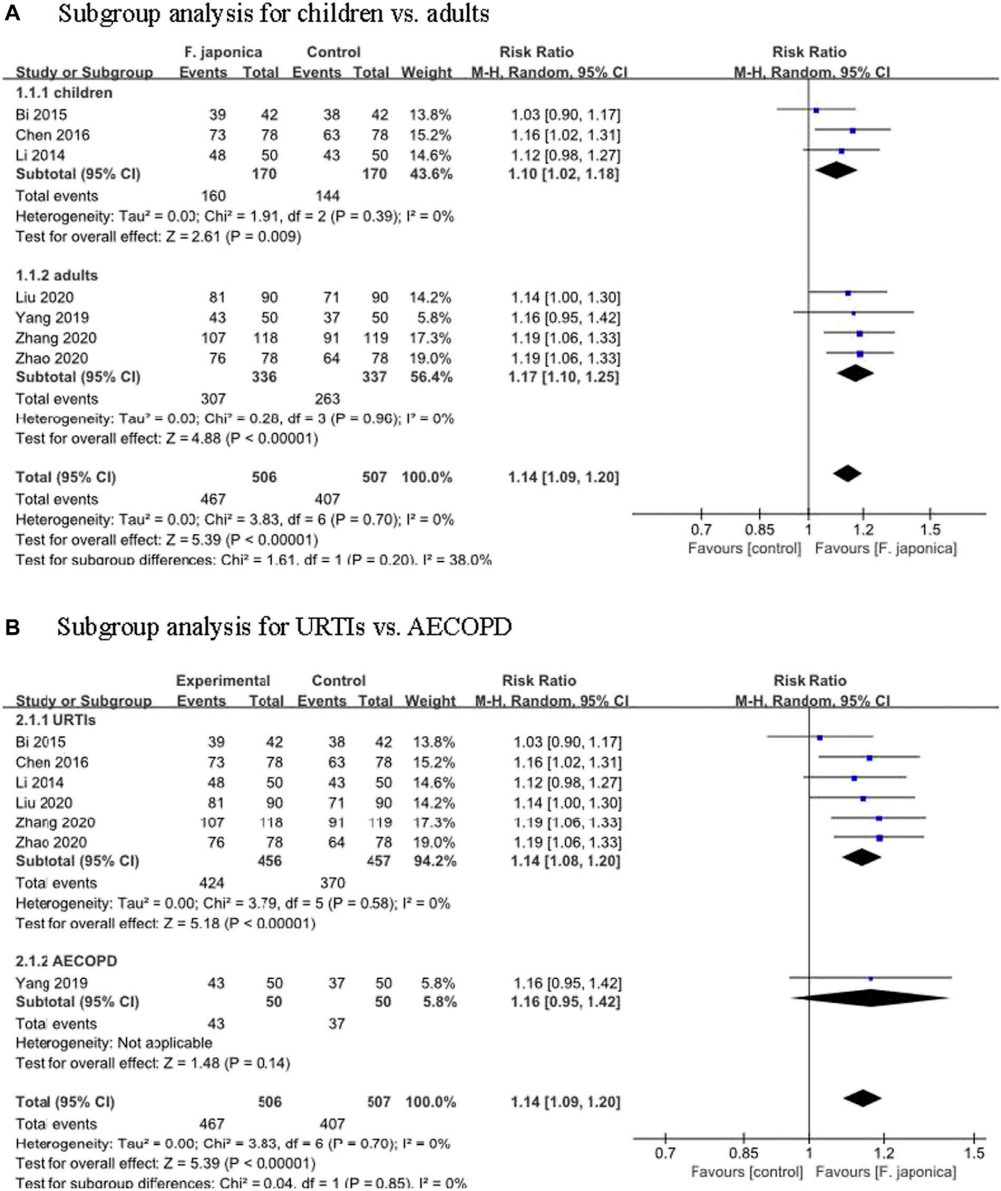
Meta-analysis of symptom improvement rate **(A)**. Subgroup analysis for children vs. adults **(B)**. Subgroup analysis for URTIs vs. AECOPD.

Symptom improvement rate was defined as (total number of patients—the number of patients with ineffective treatment)/total number of patients × 100%. The clinical effective of this systematic review was defined following symptom improvement rate, and the higher rate meant the better clinical effective. Seven RCTs (Li et al., 2014; Bi and Feng, 2015; Chen et al., 2016; Yang et al., 2019; Liu et al., 2020; Zhao and Wang, 2020; Zhang et al., 2021) evaluated the symptom improvement rate, and the meta-analysis result showed that herbal remedies that included *F. japonica* had a positive effect on symptom improvement rate when compared to usual care or usual care plus herbal remedy placebo (RR 1.14, 95% CI [1.09, 1.20], I^2^ = 0%, *p* < 0.00001, *n* = 7 trials, 1,013 participants). Similar results were found in the subgroups: for children (RR 1.10, 95% CI [1.02, 1.18], I^2^ = 0%, *p* < 0.00001, *n* = 3 trials, 340 participants) and for adults (RR 1.17, 95% CI [1.10, 1.25], I^2^ = 0%, *p* < 0.00001, *n* = 4 trials, 673 participants) (Figure 3A). Subgroups analysis for different symptoms: for URTIs, herbal remedies that included *F. japonica* had a positive effect on symptom improvement rate when compared to usual care or usual care plus herbal remedy placebo (RR 1.14, 95% CI [1.08, 1.20], I^2^ = 0%, *p* < 0.00001, *n* = 6 trials, 913 participants), while for acute exacerbations of chronic obstructive pulmonary disease (AECOPD), there was no significant difference between herbal remedies that included *F. japonica* and usual care (RR 1.16, 95% CI [0.95, 1.42], *n* = 1 trial, 100 participants) (Figure 3B).

#### 3.3.2Fever Resolution Time

Two RCTs (Bi and Feng, 2015; Chen et al., 2016) assessed fever resolution time in children, and one (Zhao and Wang, 2020) in adults. Two of the four (Bi and Feng, 2015; Chen et al., 2016) compared Shufeng Jiedu capsule plus usual care with usual care for paediatric upper RTIs: the duration was 3 days in Bi’s study and the results showed Shufeng Jiedu capsule plus usual care shortened the time with fever (MD -0.60 days, 95% CI [-0.77, -0.43]); the duration in Chen’s study was 7 days and the results showed similar findings (MD -1.70 days, 95% CI [-2.13, -1.27]). One trial (Zhao and Wang, 2020) which compared usual care plus Shufeng Jiedu capsule with usual care only, found that Shufeng Jiedu capsule shortened fever clearance time for adults (MD -1.39 days, 95% CI [-1.57, -1.21]). Overall significant differences were observed in time without fever.

#### 3.3.3 Murray Lung Injury Score

Three RCTs (Yang et al., 2019; Yang, 2019; Liu et al., 2020) evaluated the lung injury severity based simply on oxygenation criteria (PaO2/FiO2) (by calculating with PaO2/FiO2 when the fresh gas flow was off at the time of the arterial blood gas sampling, a higher score means lower percentage oxygenation and more severe lung injury) (Ntoumenopoulos et al., 2021). Those three trials focused on adults and compared herbal remedies that included *F. japonica* plus usual care with usual care: Shufeng Jiedu capsule could significantly reduce the score in acute RTIs (MD -3.49, 95% CI [-3.96 to -3.03]) (Liu et al., 2020) and in acute exacerbation of chronic obstructive pulmonary disease (AECOPD) (MD -3.49, 95% CI [-3.96 to -3.03]) (Yang et al., 2019); and Xuanfei Quyu Tongluo liquid was less likely to reduce the score in AECOPD than usual care (MD -0.60, 95% CI [-0.98 to -0.22]) (Yang Q 2019).

#### 3.3.4 Adverse Events

**FIGURE 4:**
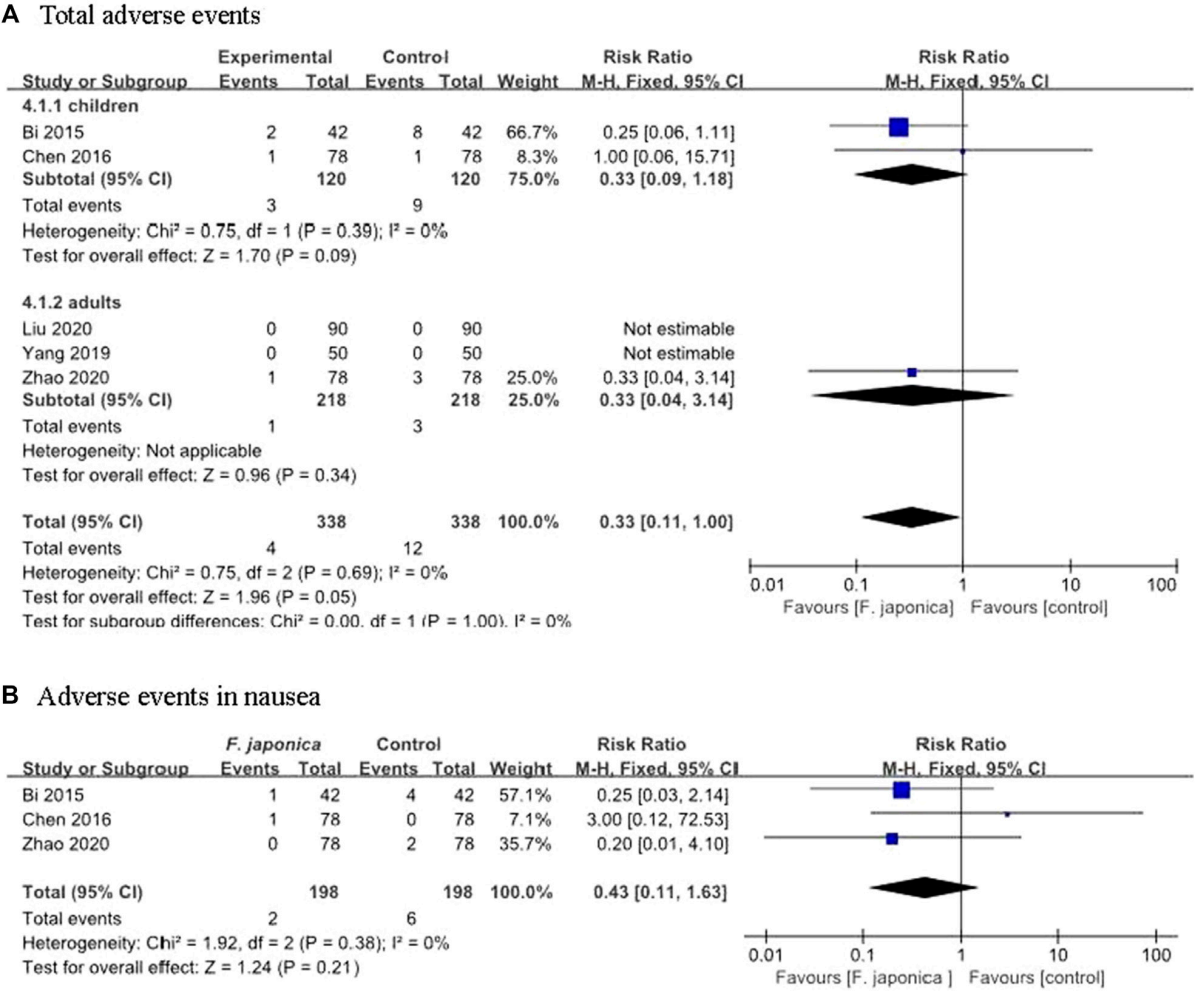
Adverse events from the including trials **(A)**. Total adverse events **(B)**. Adverse events in nausea.

Five RCTs (Bi and Feng, 2015; Chen et al., 2016; Yang, 2019; Liu et al., 2020; Zhao and Wang, 2020) assessed the adverse events of herbal remedies that included *F. japonica*. The results showed that there was no significant difference between herbal remedies that included *F. japonica* and usual care or usual care plus herbal remedy placebo (RR 0.33, 95% CI [0.11, 1.00], I^2^ = 0%, *p* = 0.05, n = 5 trials, 676 participants) (Figure 4A). One (Bi and Feng, 2015) reported two AEs (one nausea and one rash), one (Chen et al., 2016) reported one case of nausea and one (Zhao and Wang, 2020) reported one case of diarrhoea after taking Shufeng Jiedu capsule. The similar results showed that there was no significant difference between herbal remedies containing *F. japonica* (such as Shufeng Jiedu capsule) and usual care with regard to nausea (RR 0.43, 95% CI [0.11, 1.63], I^2^ = 0%, *p* = 0.21, *n* = 3 trials, 396 participants) (Figure 4B), rash (RR 0.43, 95% CI [0.06, 2.84], I^2^ = 0%, *p* = 0.38, *n* = 2 trials, 240 participants), and diarrhoea (RR 0.67, 95% CI [0.11, 3.93], I^2^ = 0%, *p* = 0.65, *n* = 2 trials, 240 participants).

#### 3.4 Evidence Level Based on GRADE

Based on the results from GRADE, we found very low quality evidence on herbal remedies that included *F. japonica* for symptom improvement rate and reducing adverse events. The reasons for down-grading were an inadequate method for random sequence generation in Bi’s study, lack of information on inconsistency, and poor publication quality of all the included trials (Supplementary Appendix S2).

## 4 Discussion

The effectiveness and safety of *F. japonica*, which is widely used for infectious diseases such as RTIs, were evaluated in this systematic review. The results show some evidence of benefit with herbal remedies that included *F. japonica* for acute RTIs in adults and children, although the quality of all included trials was rated as poor. There were no studies on *F. japonica* as a monotherapy for acute RTIs.

### 4.1 Summary of the Main Results

#### 4.1.1 *F. japonica* as a Monotherapy for RTIs

The aim of this systematic review focused on evaluating *F. japonica* for RTIs, but no trials used it as a monotherapy in the present evidence. So we could not draw any conclusions about the effectiveness of *F. japonica* as a monotherapy for improving symptoms of RTIs.

### 4.1.2 *F. japonica* in Herbal Remedies for RTIs

After combining the included eight RCTs in this systematic review, the results showed that patients with acute RTIs who took *F. japonica* in a herbal remedy had faster rates of symptom improvement regardless of age. Also, *F. japonica* in a herbal mixture could reduce the duration of fever and the Murray lung injury score when comparing the herbal remedy plus usual care with usual care alone, or comparing the herbal remedy alone with usual care. For adverse events, there were no statistically significant differences between herbal remedies that included *F. japonica* and usual care. In conclusion, *F. japonica* as part of a multi herbal remedy might be an effective and safe option for acute RTIs. Only one included trial compared herbal medicine (Tuire Liquid) to usual care; all the others used herbal medicine plus usual care including antibiotics.

### 4.2 Previous Studies on *F. japonica* for RTIs

To our best knowledge, there has been no previous meta-analysis of clinical trials of *F. japonica* for acute RTIs. It has always been used in combination with other herbs in clinical practice: in this review, Shufeng Jiedu capsule and Qingfei liquid were the most commonly used remedies that included *F. japonica* for RTIs. Although the mechanism of *F. japonica* for RTIs is still unclear, herbal remedies treating RTIs have multiple possible active compounds, mechanisms of action, targets and pathways (Xu et al., 2020). The findings from our previous systematic review verified that Shufeng Jiedu capsule could be a therapeutic option for shortening the duration of the typical symptoms in acute URTIs without serious adverse events (Zhang et al., 2021); this previous study showed similar results to this systematic review.

### 4.3 Limitations

This systematic review had some limitations. Although comprehensive searches were carried out on 12 databases, this review may have missed some trials: potentially eligible trials might be missed if the duration of the condition was not reported, therefore patients with acute RTIs could not be identified, or the original trial assessed a complex herbal remedy which did not properly index *F. japonica* despite its inclusion in the remedy. All included RCTs used *F. japonica* in a multi-herbal formula, so we could not evaluate the effect of this herb alone for RTIs. All the included RCTs in this review were carried out in China and published in Chinese, which may lead to language bias and may lack generalizability of the results from this review. All included studies showed positive results for herbal remedies that included *F. japonica* therapy, but there may be publication bias because some negative results may not be published in peer-reviewed literature. Only one (12.5%) of the included trials used a herbal placebo. Finally, all included trials lacked information on their protocol or registered outcomes. Overall the quality of the included studies was rated as low, and so the results of the review should be regarded with caution.

### 4.4 Implications for Future Research

All the interventions in the included RCTs comprised *F. japonica* as part of a herbal formula, while no trial examined the use of *F. japonica* as a monotherapy to treat acute RTIs. Herbal remedies with *F. japonica* appear to have helpful effects in relief of RTI symptoms. Therefore, we suggest *F. japonica* in a herbal remedy could be useful in treating RTIs. *F. japonica* acts as the sovereign drug in some herbal remedies such as Shufeng Jiedu capsule for RTIs, so we suggest *F. japonica* may possibly have efficacy as a single intervention for RTIs, but more RCTs of better quality need to be conducted. High quality trials with precise methodological design and rigorous reporting on the evaluation of *F. japonica* for acute RTIs should be carried out. Using appropriate methods of random allocation, blinding, estimating the sample size of participants, and developing a detailed study protocol should be promoted for future relevant clinical trials to ensure high-quality. Future studies could compare *F. japonica* with placebo and report the details on blinding for participants, investigators and outcome assessors. Since nearly all the studies were carried out in China, multi-centre or international studies could be conducted. Children are a special population and the safety of children’s medication should be critical, so the safety assessment for children should be given attention. The most important thing for future clinical trials is evaluating the effectiveness and safety of using herbal medicine without antibiotics for RTIs, or measuring antibiotic use reduction as a main outcome measure, which could really contribute to reducing the use of antibiotics.

## 5 Conclusion

There is limited but some evidence that *F. japonica* as part of an herbal mixture may be an effective and safe intervention for acute RTIs in clinical practice. Nevertheless, the findings in this review should be interpreted with caution due to the limited methodological quality of the included RCTs. It is better to evaluate the effectiveness and safety of using herbal medicine without antibiotics for acute RTIs in future studies.

## Data Availability

The original contributions presented in the study are included in the article/Supplementary Material further inquiries can be directed to the corresponding authors.

## Abbreviations

AECOPD, acute exacerbation of chronic obstructive pulmonary disease; AMED, Allied and Complementary Medicine Database; ARI, acute respiratory infection; CI, confidence intervals; CHM, Chinese herbal medicine; CNKI, China National Knowledge Infrastructure; COPD, chronic obstructive pulmonary disease; FDA, the Food and Drug Administration; *F. japonica*, *Reynoutria japonica* Houtt; GRADE, Grades of Recommendations Assessment, Development and Evaluation; ITT, intention to treat; MD, mean differences; MMP-9, matrix metalloproteinase-9; NF-Κb, κ-light chain of nuclear factor B cells; OASIS, Oriental Medicine Advanced Searching Integrated System; PD, Polydatin; PRISMA, the Preferred Reporting Items for Systematic Reviews and Meta-Analyses; RTIs, Respiratory tract infections; RISS, Sino-Med Database Research Information Service System; RCTs, randomized controlled trials; RR, risk ratio; SM, standardized mean differences; TCM, Traditional Chinese Medicine; TIMP-1, tissue inhibitor of metalloprotease-1; VIP, Chinese Science and Technology Journal Database; WHO, the World Health Organization.
