# Supplementary material for: Reynoutria japonica Houtt for Acute Respiratory Tract Infections in Adults and Children: A Systematic Review
Source: Front Pharmacol. 2022 Feb 24;13:787032. doi: 10.3389/fphar.2022.787032 (PMC8911541; doi:10.3389/fphar.2022.787032)
Supplement: Supplementary file 1 [file DataSheet2.docx]

Appendix 2 Evidence level based on GRADE

| **Patient or population:** patients with RITs **Settings:** RCTs **Intervention:** herbal remedies that included F. japonica | | | | | | |
| --- | --- | --- | --- | --- | --- | --- |
| **Outcomes** | **Illustrative comparative risks* (95% CI)** | | **Relative effect (95% CI)** | **No of Participants (studies)** | **Quality of the evidence (GRADE)** | **Comments** |
|  | Assumed risk | Corresponding risk |  |  |  |  |
|  | **Control** | **Herbal remedies that included F. japonica** |  |  |  |  |
| **Symptom improvement rate** clinical effective Follow-up: 3-7 days | **Study population** | | **RR 1.14**  (1.09 to 1.20) | 1013 (7 studies) | ⊕⊝⊝⊝ **very low**^1,2,3^ |  |
|  | **803 per 1000** | **915 per 1000** (875 to 963) |  |  |  |  |
|  | **Moderate** | |  |  |  |  |
|  |  |  |  |  |  |  |
| **Adverse events**  number of the AEs Follow-up: 3-7 days | **Study population** | | **RR 0.33**  (0.11 to 1.00) | 676 (5 studies) | ⊕⊝⊝⊝ **very low**^1,2,3^ |  |
|  | **36 per 1000** | **12 per 1000** (4 to 36) |  |  |  |  |
|  | **Moderate** | |  |  |  |  |
|  |  |  |  |  |  |  |
| *The basis for the **assumed risk** (e.g. the median control group risk across studies) is provided in footnotes. The **corresponding risk** (and its 95% confidence interval) is based on the assumed risk in the comparison group and the **relative effect** of the intervention (and its 95% CI). **CI:** Confidence interval; **RR:** Risk ratio; | | | | | | |
| GRADE Working Group grades of evidence **High quality:** Further research is very unlikely to change our confidence in the estimate of effect.  **Moderate quality:** Further research is likely to have an important impact on our confidence in the estimate of effect and may change the estimate. **Low quality:** Further research is very likely to have an important impact on our confidence in the estimate of effect and is likely to change the estimate. **Very low quality:** We are very uncertain about the estimate. | | | | | | |
| ^1^ Bi 2015 reported a wrong method for random sequence generation.  ^2^ Lack of the information in all included trials. ^3^ Poor quality of all included trials according to RoB. | | | | | | |
