# Supplementary material for: Reynoutria japonica Houtt for Acute Respiratory Tract Infections in Adults and Children: A Systematic Review
Source: Front Pharmacol. 2022 Feb 24;13:787032. doi: 10.3389/fphar.2022.787032 (PMC8911541; doi:10.3389/fphar.2022.787032)
Supplement: Supplementary file 2 [file DataSheet1.docx]

**Appendix 1**

**Medline (PubMed): From inception to 31^st^ July 2021**

1. exp Respiratory Tract Infections/

2. (respiratory tract infection* or (respiratory adj3 infection*) or RTI* or (chest adj3 infection*) or upper respiratory tract infection* or upper respiratory infection* or lower respiratory tract infection* or lower respiratory infection*).mp.

3. exp Tonsillitis/

4. exp Rhinitis/

5. exp Sinusitis/

6. exp Pharyngitis/

7. exp Laryngitis/

8. exp Nasopharyngitis/

9. (tonsillit* or rhinit* or sinusit* or pharyngit* or laryngit* or rhinosinusit* or rhinopharyngit* or rhinolaryngit* or nasosinusit* or nasopharyngit* or nasolaryngit* or sinonasal* or

rhino-sinusit* or rhino-pharyngit* or rhino-laryngit* or naso-sinusit* or naso-pharyngit* or sino-nasal* or ear* or otit*).mp.

10. exp Bronchitis/

11. exp Supraglottitis/

12. exp Tracheitis/

13. (bronchit* or supraglottit* or epiglott* or pulmon* or tracheit* or respirat*).mp.

14. exp cough/

15. exp common cold/

16. exp influenza, human/

17. exp Croup/

18. (cough* or cold* or catarrh or flu or influenza or (sore adj3 throat) or (throat adj3 pain) or (blocked adj3 nose) or (runn* adj3 nose) or (stuff* adj3 nose) or (short* adj3 breath*) or rhinorrh?ea or congest* or discharge* or phlegm*).mp.

19. exp Pulmonary Disease, Chronic Obstructive/

20. exp Asthma/

21. (acute exacerbation of COPD* or ae COPD* or acute exacerbation of chronic obstructive pulmonary disease* or acute exacerbation of asthma or acute asthma or acute asthma exacerbation or asthma exacerbation* or dyspnea* or bronchoconstrict* or bronchial constrict* or bronch* or bronchospas* or obstruct* adj3 (pulmon* or lung or airway* or airflow*)).mp.

22. or/1-21

23. Fallopia/

24. (*Fallopia japonica* (Houtt.) or Giant Knotweed or Mexican Bamboo or Kontiki Bamboo or Japanese Bamboo or Japanese Knotweed or Asian Knotweed or Asiatic Knotweed or Japanese Fleece Flower or Donkey Rhubarb or Parkslide or Itadori or Persicaire Cuspidée or *Fallopia* *japonica* f. *colorans* (Makino) or *Fallopia compacta* (Hook) or *Fallopia* *japonica* var. *hachidyoensis* (Makino) or *Fallopia* *japonica* var. *uzenensis* (Honda) or *Pleuropterus* *cuspidatus* (Siebold & Zucc.) or *Pleuropterus* *zuccarinii* (Small) or *Reynoutria japonica* or *Reynoutria* *hachidyoensis* (Makino) Nakai or *Reynoutria* *compacta* (Hook.f.) Nakai or *Reynoutria* *hastata* Nakai ex Ui or *Reynoutria* *henryi* Nakai or *Reynoutria* *uzenensis* (Honda) Honda or *Tiniaria* *japonica* (Houtt.) Hedberg or *Polygonum cuspidatum* or *Polygonum compactum* (Hook) or *Polygonum* *sieboldii* de Vriese ex L.H.Bailey or *Polygonum reynoutria* Makino or *Polygonum* *reynoutria* f. *colorans* Makino or *Polygonum zuccarinii* Small or *Polygonum reynoutria* f. *colorans* Makino or *Polygonum hachidyoense* Makino or Rhizoma et radix polygoni cuspidate).mp.

25. (Hu-Zhang or 虎杖 or Kuzhang or Banzhang or Suantongsun or Bangen or Suantanggan or Suangan or Zijinlong or Huoxuelong or Huoxuedan or Yinyanglian or Daye Shezongguan or Jiulonggen or Huangdiyu or Dachongzhang or Bancao or Jiulonggen or Shanqiezi or Bandaozeng or Banzhuang or Banzhuanggen or Wuzhang or Kuzhang or Niaobuta or Banlongzi or Yehuanglian or Shujinlong or Gangyagen or Dahuoxue or Xueteng or Haotongcao or Hongguanjiao or Daganshu or Taichongzhang or Ganchugen or Huabanzhu or Yeshezongguan).mp.

26. or/23-25

27. 22 and 26

Limit 27 to human(s)

*=truncation, exp=explode, adj3=adjacent within 3 words of each other in either direction, ?=substitute for one or no characters

mp=title, abstract, original title, name of substance word, subject heading word, keyword heading word, protocol supplementary concept word, rare disease supplementary concept word, unique identifier

**OMID: AMED From 1985 to 31^st^ July 2021**

1. exp Respiratory Tract Infections/

2. (respiratory tract infection* or (respiratory adj3 infection*) or RTI* or (chest adj3 infection*) or upper respiratory tract infection* or upper respiratory infection* or lower respiratory tract infection* or lower respiratory infection*).mp.

3. exp Tonsillitis/

4. exp Rhinitis/

5. exp Sinusitis/

6. exp Pharyngitis/

7. exp Laryngitis/

8. exp Nasopharyngitis/

9. (tonsillit* or rhinit* or sinusit* or pharyngit* or laryngit* or rhinosinusit* or rhinopharyngit* or rhinolaryngit* or nasosinusit* or nasopharyngit* or nasolaryngit* or sinonasal* or

rhino-sinusit* or rhino-pharyngit* or rhino-laryngit* or naso-sinusit* or naso-pharyngit* or sino-nasal* or ear* or otit*).mp.

10. exp Bronchitis/

11. exp Supraglottitis/

12. exp Tracheitis/

13. (bronchit* or supraglottit* or epiglott* or pulmon* or tracheit* or respirat*).mp.

14. exp cough/

15. exp common cold/

16. exp influenza, human/

17. exp Croup/

18. (cough* or cold* or catarrh or flu or influenza or (sore adj3 throat) or (throat adj3 pain) or (blocked adj3 nose) or (runn* adj3 nose) or (stuff* adj3 nose) or (short* adj3 breath*) or rhinorrh?ea or congest* or discharge* or phlegm*).mp.

19. exp Pulmonary Disease, Chronic Obstructive/

20. exp Asthma/

21. (acute exacerbation of COPD* or ae COPD* or acute exacerbation of chronic obstructive pulmonary disease* or acute exacerbation of asthma or acute asthma or acute asthma exacerbation or asthma exacerbation* or dyspnea* or bronchoconstrict* or bronchial constrict* or bronch* or bronchospas* or obstruct* adj3 (pulmon* or lung or airway* or airflow*)).mp.

22. or/1-21

23. Fallopia/

24. (*Fallopia japonica* (Houtt.) or Giant Knotweed or Mexican Bamboo or Kontiki Bamboo or Japanese Bamboo or Japanese Knotweed or Asian Knotweed or Asiatic Knotweed or Japanese Fleece Flower or Donkey Rhubarb or Parkslide or Itadori or Persicaire Cuspidée or *Fallopia* *japonica* f. *colorans* (Makino) or *Fallopia compacta* (Hook) or *Fallopia* *japonica* var. *hachidyoensis* (Makino) or *Fallopia* *japonica* var. *uzenensis* (Honda) or *Pleuropterus* *cuspidatus* (Siebold & Zucc.) or *Pleuropterus* *zuccarinii* (Small) or *Reynoutria japonica* or *Reynoutria* *hachidyoensis* (Makino) Nakai or *Reynoutria* *compacta* (Hook.f.) Nakai or *Reynoutria* *hastata* Nakai ex Ui or *Reynoutria* *henryi* Nakai or *Reynoutria* *uzenensis* (Honda) Honda or *Tiniaria* *japonica* (Houtt.) Hedberg or *Polygonum cuspidatum* or *Polygonum compactum* (Hook) or *Polygonum* *sieboldii* de Vriese ex L.H.Bailey or *Polygonum reynoutria* Makino or *Polygonum* *reynoutria* f. *colorans* Makino or *Polygonum zuccarinii* Small or *Polygonum reynoutria* f. *colorans* Makino or *Polygonum hachidyoense* Makino or Rhizoma et radix polygoni cuspidate).mp.

25. (Hu-Zhang or 虎杖 or Kuzhang or Banzhang or Suantongsun or Bangen or Suantanggan or Suangan or Zijinlong or Huoxuelong or Huoxuedan or Yinyanglian or Daye Shezongguan or Jiulonggen or Huangdiyu or Dachongzhang or Bancao or Jiulonggen or Shanqiezi or Bandaozeng or Banzhuang or Banzhuanggen or Wuzhang or Kuzhang or Niaobuta or Banlongzi or Yehuanglian or Shujinlong or Gangyagen or Dahuoxue or Xueteng or Haotongcao or Hongguanjiao or Daganshu or Taichongzhang or Ganchugen or Huabanzhu or Yeshezongguan).mp.

26. or/23-25

27. 22 and 26

Limit 27 to human(s)

*=truncation, exp=explode, adj3=adjacent within 3 words of each other in either direction, ?=substitute for one or no characters

mp=title, abstract, original title, name of substance word, subject heading word, keyword heading word, protocol supplementary concept word, rare disease supplementary concept word, unique identifier

**Ovid: Embase From 1947 to 31^st^ July 2021**

1. exp Respiratory Tract Infections/

2. (respiratory tract infection* or (respiratory adj3 infection*) or RTI* or (chest adj3 infection*) or upper respiratory tract infection* or upper respiratory infection* or lower respiratory tract infection* or lower respiratory infection*).mp.

3. exp Tonsillitis/

4. exp Rhinitis/

5. exp Sinusitis/

6. exp Pharyngitis/

7. exp Laryngitis/

8. exp Nasopharyngitis/

9. (tonsillit* or rhinit* or sinusit* or pharyngit* or laryngit* or rhinosinusit* or rhinopharyngit* or rhinolaryngit* or nasosinusit* or nasopharyngit* or nasolaryngit* or sinonasal* or

rhino-sinusit* or rhino-pharyngit* or rhino-laryngit* or naso-sinusit* or naso-pharyngit* or sino-nasal* or ear* or otit*).mp.

10. exp Bronchitis/

11. exp Supraglottitis/

12. exp Tracheitis/

13. (bronchit* or supraglottit* or epiglott* or pulmon* or tracheit* or respirat*).mp.

14. exp cough/

15. exp common cold/

16. exp influenza, human/

17. exp Croup/

18. (cough* or cold* or catarrh or flu or influenza or (sore adj3 throat) or (throat adj3 pain) or (blocked adj3 nose) or (runn* adj3 nose) or (stuff* adj3 nose) or (short* adj3 breath*) or rhinorrh?ea or congest* or discharge* or phlegm*).mp.

19. exp Pulmonary Disease, Chronic Obstructive/

20. exp Asthma/

21. (acute exacerbation of COPD* or ae COPD* or acute exacerbation of chronic obstructive pulmonary disease* or acute exacerbation of asthma or acute asthma or acute asthma exacerbation or asthma exacerbation* or dyspnea* or bronchoconstrict* or bronchial constrict* or bronch* or bronchospas* or obstruct* adj3 (pulmon* or lung or airway* or airflow*)).mp.

22. or/1-21

23. Fallopia/

24. (*Fallopia japonica* (Houtt.) or Giant Knotweed or Mexican Bamboo or Kontiki Bamboo or Japanese Bamboo or Japanese Knotweed or Asian Knotweed or Asiatic Knotweed or Japanese Fleece Flower or Donkey Rhubarb or Parkslide or Itadori or Persicaire Cuspidée or *Fallopia* *japonica* f. *colorans* (Makino) or *Fallopia compacta* (Hook) or *Fallopia* *japonica* var. *hachidyoensis* (Makino) or *Fallopia* *japonica* var. *uzenensis* (Honda) or *Pleuropterus* *cuspidatus* (Siebold & Zucc.) or *Pleuropterus* *zuccarinii* (Small) or *Reynoutria japonica* or *Reynoutria* *hachidyoensis* (Makino) Nakai or *Reynoutria* *compacta* (Hook.f.) Nakai or *Reynoutria* *hastata* Nakai ex Ui or *Reynoutria* *henryi* Nakai or *Reynoutria* *uzenensis* (Honda) Honda or *Tiniaria* *japonica* (Houtt.) Hedberg or *Polygonum cuspidatum* or *Polygonum compactum* (Hook) or *Polygonum* *sieboldii* de Vriese ex L.H.Bailey or *Polygonum reynoutria* Makino or *Polygonum* *reynoutria* f. *colorans* Makino or *Polygonum zuccarinii* Small or *Polygonum reynoutria* f. *colorans* Makino or *Polygonum hachidyoense* Makino or Rhizoma et radix polygoni cuspidate).mp.

25. (Hu-Zhang or 虎杖 or Kuzhang or Banzhang or Suantongsun or Bangen or Suantanggan or Suangan or Zijinlong or Huoxuelong or Huoxuedan or Yinyanglian or Daye Shezongguan or Jiulonggen or Huangdiyu or Dachongzhang or Bancao or Jiulonggen or Shanqiezi or Bandaozeng or Banzhuang or Banzhuanggen or Wuzhang or Kuzhang or Niaobuta or Banlongzi or Yehuanglian or Shujinlong or Gangyagen or Dahuoxue or Xueteng or Haotongcao or Hongguanjiao or Daganshu or Taichongzhang or Ganchugen or Huabanzhu or Yeshezongguan).mp.

26. or/23-25

27. 22 and 26

Limit 27 to human(s)

*=truncation, exp=explode, adj3=adjacent within 3 words of each other in either direction, ?=substitute for one or no characters

mp=title, abstract, original title, name of substance word, subject heading word, keyword heading word, protocol supplementary concept word, rare disease supplementary concept word, unique identifier

**CINAHL Plus with Full Text (EBSCO): From 1937 to 31^st^ July 2021**

1. (MH "Respiratory Tract Infections+")

2. TI respiratory tract infection* or AB respiratory tract infection* or SU respiratory tract infection* or TI (respiratory N3 infection*) or AB (respiratory N3 infection*) or SU (respiratory N3 infection*) or TI RTI* or AB RTI* or SU RTI* or TI (chest N3 infection*) or AB (chest N3 infection*) or SU (chest N3 infection*)

3. TI (tonsilit* or rhinit* or sinusit* or pharyngit* or laryngit* or rhinosinusit* or rhinopharyngit* or rhinolaryngit* or nasosinusit* or nasopharyngit* or nasolaryngit* or sinonasal* or rhino-sinusit* or rhino-pharyngit* or rhino-laryngit* or naso-sinusit* or naso-pharyngit* or sino-nasal* or ear* or otit*) or AB (rhinit* or sinusit* or pharyngit* or laryngit* or rhinosinusit* or rhinopharyngit* or rhinolaryngit* or nasosinusit* or nasopharyngit* or nasolaryngit* or sinonasal* or rhino-sinusit* or rhino-pharyngit* or rhino-laryngit* or naso-sinusit* or naso-pharyngit* or sino-nasal* or ear* or otit*) or SU (rhinit* or sinusit* or pharyngit* or laryngit* or rhinosinusit* or rhinopharyngit* or rhinolaryngit* or nasosinusit* or nasopharyngit* or nasolaryngit* or sinonasal* or rhino-sinusit* or rhino-pharyngit* or rhino-laryngit* or naso-sinusit* or naso-pharyngit* or sino-nasal* or ear* or otit*)

4. TI (bronchit* or supraglottit* or epiglott* or pulmon* or tracheit* or respirat*) or AB (bronchit* or supraglottit* or epiglott* or pulmon* or tracheit* or respirat*) or SU (bronchit* or supraglottit* or epiglott* or pulmon* or tracheit* or respirat*)

5. TI (cough* or croup* or cold* or catarrh or flu or influenza or (sore adj3 throat) or (throat adj3 pain) or (blocked adj3 nose) or (runn* adj3 nose) or (stuff* adj3 nose) or (short* adj3 breath*) or rhinorrh?ea or congest* or discharge*) or AB (cough* or croup* or cold* or catarrh or flu or influenza or (sore adj3 throat) or (throat adj3 pain) or (blocked adj3 nose) or (runn* adj3 nose) or (stuff* adj3 nose) or (short* adj3 breath*) or rhinorrh?ea or congest* or discharge*) or SU (cough* or croup* or cold* or catarrh or flu or influenza or (sore adj3 throat) or (throat adj3 pain) or (blocked adj3 nose) or (runn* adj3 nose) or (stuff* adj3 nose) or (short* adj3 breath*) or rhinorrh?ea or congest* or discharge*)

6. TI ((cough* or croup* or cold* or catarrh or flu or influenza or (sore N3 throat) or (throat N3 pain) or (blocked N3 nose) or (runn* N3 nose) or (stuff* N3 nose) or (short* N3 breath*) or rhinorrh?ea or congest* or discharge*)) or AB ((cough* or croup* or cold* or catarrh or flu or influenza or (sore N3 throat) or (throat N3 pain) or (blocked N3 nose) or (runn* N3 nose) or (stuff* N3 nose) or (short* N3 breath*) or rhinorrh?ea or congest* or discharge*)) or SU ((cough* or croup* or cold* or catarrh or flu or influenza or (sore N3 throat) or (throat N3 pain) or (blocked N3 nose) or (runn* N3 nose) or (stuff* N3 nose) or (short* N3 breath*) or rhinorrh?ea or congest* or discharge*))

7. (MH "Acute Exacerbations Chronic Obstructive Pulmonary Disease+")

8. (MH “acute exacerbations of asthma+”)

9. TI (obstruct* adj3 (pulmon* or lung or airway* or airflow*) or dyspnea* or bronchoconstrict* or bronchial constrict* or bronch* or bronchospas* or AB (obstruct* adj3 (pulmon* or lung or airway* or airflow*) or dyspnea* or bronchoconstrict* or bronchial constrict* or bronch* or bronchospas*) or SU (obstruct* adj3 (pulmon* or lung or airway* or airflow*) or dyspnea* or bronchoconstrict* or bronchial constrict* or bronch* or bronchospas* )

10. or/1-9

11. TI (*Fallopia japonica* (Houtt.) or Giant Knotweed or Mexican Bamboo or Kontiki Bamboo or Japanese Bamboo or Japanese Knotweed or Asian Knotweed or Asiatic Knotweed or Japanese Fleece Flower or Donkey Rhubarb or Parkslide or Itadori or Persicaire Cuspidée or *Fallopia* *japonica* f. *colorans* (Makino) or *Fallopia compacta* (Hook) or *Fallopia* *japonica* var. *hachidyoensis* (Makino) or *Fallopia* *japonica* var. *uzenensis* (Honda) or *Pleuropterus* *cuspidatus* (Siebold & Zucc.) or *Pleuropterus* *zuccarinii* (Small) or *Reynoutria japonica* or *Reynoutria* *hachidyoensis* (Makino) Nakai or *Reynoutria* *compacta* (Hook.f.) Nakai or *Reynoutria* *hastata* Nakai ex Ui or *Reynoutria* *henryi* Nakai or *Reynoutria* *uzenensis* (Honda) Honda or *Tiniaria* *japonica* (Houtt.) Hedberg or *Polygonum cuspidatum* or *Polygonum compactum* (Hook) or *Polygonum* *sieboldii* de Vriese ex L.H.Bailey or *Polygonum reynoutria* Makino or *Polygonum* *reynoutria* f. *colorans* Makino or *Polygonum zuccarinii* Small or *Polygonum reynoutria* f. *colorans* Makino or *Polygonum hachidyoense* Makino or Rhizoma et radix polygoni cuspidate or Hu-Zhang or 虎杖 or Kuzhang or Banzhang or Suantongsun or Bangen or Suantanggan or Suangan or Zijinlong or Huoxuelong or Huoxuedan or Yinyanglian or Daye Shezongguan or Jiulonggen or Huangdiyu or Dachongzhang or Bancao or Jiulonggen or Shanqiezi or Bandaozeng or Banzhuang or Banzhuanggen or Wuzhang or Kuzhang or Niaobuta or Banlongzi or Yehuanglian or Shujinlong or Gangyagen or Dahuoxue or Xueteng or Haotongcao or Hongguanjiao or Daganshu or Taichongzhang or Ganchugen or Huabanzhu or Yeshezongguan)

12. 10 and 110

*=truncation, N3=finds the words if they are within five words of one another, regardless of the order in which they appear, ?= replaces that number of character(s)

MH=MeSH, TI=title, AB=abstract, SU=subject,

**Cochrane Central Register of Controlled Trials (CENTRAL) on the Cochrane Library: From inception to 31^st^ July 2021**

1. MeSH descriptor: [Respiratory Tract Infections] explode all trees

2. (respiratory tract infection* or (respiratory near infection*) or RTI or (chest near infection*) or upper respiratory tract infection* or upper respiratory infection* or lower respiratory tract infection* or lower respiratory infection*)

3. (tonsilit* or rhinit* or sinusit* or pharyngit* or laryngit* or rhinosinusit* or rhinopharyngit* or rhinolaryngit* or nasosinusit* or nasopharyngit* or nasolaryngit* or sinonasal* or

rhino-sinusit* or rhino-pharyngit* or rhino-laryngit* or naso-sinusit* or naso-pharyngit* or sino-nasal* or ear* or otit*)

4. (bronchit* or supraglottit* or epiglott* or pulmon* or tracheit* or respirat*)

5. (cough* or croup* or cold* or catarrh or flu or influenza or (sore near throat) or (throat near pain) or (blocked adj3 nose) or (runn* adj3 nose) or (stuff* near nose) or (short* near breath*) or rhinorrh?ea or congest* or discharge*)

6. #1 or #2 or #3 or #4 or #5

7. MeSH descriptor: [Acute Exacerbations Chronic Obstructive Pulmonary Disease] explode all trees

8. MeSH descriptor: [Acute Exacerbations Asthma] explode all trees

9. (obstruct* or pulmon* or lung or airway* or airflow* or dyspnea* or bronchoconstrict* or bronchial constrict* or bronch* or bronchospas*)

10. #7 and #8 or # 9

11. MeSH descriptor: [Fallopia] explode all trees

12. (*Fallopia japonica* (Houtt.) or Giant Knotweed or Mexican Bamboo or Kontiki Bamboo or Japanese Bamboo or Japanese Knotweed or Asian Knotweed or Asiatic Knotweed or Japanese Fleece Flower or Donkey Rhubarb or Parkslide or Itadori or Persicaire Cuspidée or *Fallopia* *japonica* f. *colorans* (Makino) or *Fallopia compacta* (Hook) or *Fallopia* *japonica* var. *hachidyoensis* (Makino) or *Fallopia* *japonica* var. *uzenensis* (Honda) or *Pleuropterus* *cuspidatus* (Siebold & Zucc.) or *Pleuropterus* *zuccarinii* (Small) or *Reynoutria japonica* or *Reynoutria* *hachidyoensis* (Makino) Nakai or *Reynoutria* *compacta* (Hook.f.) Nakai or *Reynoutria* *hastata* Nakai ex Ui or *Reynoutria* *henryi* Nakai or *Reynoutria* *uzenensis* (Honda) Honda or *Tiniaria* *japonica* (Houtt.) Hedberg or *Polygonum cuspidatum* or *Polygonum compactum* (Hook) or *Polygonum* *sieboldii* de Vriese ex L.H.Bailey or *Polygonum reynoutria* Makino or *Polygonum* *reynoutria* f. *colorans* Makino or *Polygonum zuccarinii* Small or *Polygonum reynoutria* f. *colorans* Makino or *Polygonum hachidyoense* Makino or Rhizoma et radix polygoni cuspidate or Hu-Zhang or 虎杖 or Kuzhang or Banzhang or Suantongsun or Bangen or Suantanggan or Suangan or Zijinlong or Huoxuelong or Huoxuedan or Yinyanglian or Daye Shezongguan or Jiulonggen or Huangdiyu or Dachongzhang or Bancao or Jiulonggen or Shanqiezi or Bandaozeng or Banzhuang or Banzhuanggen or Wuzhang or Kuzhang or Niaobuta or Banlongzi or Yehuanglian or Shujinlong or Gangyagen or Dahuoxue or Xueteng or Haotongcao or Hongguanjiao or Daganshu or Taichongzhang or Ganchugen or Huabanzhu or Yeshezongguan)

13. #11 or #12

14. random* (Word variations have been searched)

15. #6 and #10 and #13 and #14

*=truncation, exp=explode, adj3=adjacent within 3 words of each other in either direction, ?=substitute for one or no characters

mp=title, abstract, original title, name of substance word, subject heading word, keyword heading word, protocol supplementary concept word, rare disease supplementary concept word, unique identifier

**China Network Knowledge Infrastructure (CNKI): From inception to 31^st^ July 2021**

SU=('慢性阻塞性肺病'+'慢性阻塞性肺病急性加重'+'慢性阻塞性肺病急性发作'+'呼吸道感染'+'鼻炎'+'鼻窦炎'+'咽炎'+'喉炎'+'鼻咽炎'+'扁桃体炎'+'支气管炎'+'气管炎'+'咳嗽'+'感冒'+'外感'+'流感'+'喉咙痛'+'咽痛'+'咽喉痛'+'咽痒'+'鼻塞'+'流涕'+'咳'+'嗽'+'喘'+'肺'+'鼻'+'咽'+'喉') AND SU=('虎杖'+'苦杖'+'斑杖'+'酸桶笋'+'斑根'+'酸汤杆'+'紫金龙'+'活血龙'+'阴阳莲+'大叶蛇总管'+'九根龙'+'黄地榆'+'大虫杖'+'斑草'+'山茄子'+'搬倒甑'+'斑庄'+'斑庄根'+'武杖'+'鸟不踏'+'酸干'+'斑龙紫'+'野黄连'+'活血丹'+'舒筋龙'+'刚压根'+'大活血'+'血藤'+'号筒草'+'红贯脚'+'酒虎杖'+'大绀著'+'太虫杖'+'甘除根'+'酸桶草'+'酸筒草'+'花斑竹'+'刚连根'+'叶蛇总管')

SU= subject heading

**Chinese Scientific Journals Database (VIP): From inception to 31^st^ July 2021**

R=('慢性阻塞性肺病'+'慢性阻塞性肺病急性加重'+'慢性阻塞性肺病急性发作'+'呼吸道感染'+'鼻炎'+'鼻窦炎'+'咽炎'+'喉炎'+'鼻咽炎'+'扁桃体炎'+'支气管炎'+'气管炎'+'咳嗽'+'感冒'+'外感'+'流感'+'喉咙痛'+'咽痛'+'咽喉痛'+'咽痒'+'鼻塞'+'流涕'+'咳'+'嗽'+'喘'+'肺'+'鼻'+'咽'+'喉') * R=('虎杖'+'苦杖'+'斑杖'+'酸桶笋'+'斑根'+'酸汤杆'+'紫金龙'+'活血龙'+'阴阳莲+'大叶蛇总管'+'九根龙'+'黄地榆'+'大虫杖'+'斑草'+'山茄子'+'搬倒甑'+'斑庄'+'斑庄根'+'武杖'+'鸟不踏'+'酸干'+'斑龙紫'+'野黄连'+'活血丹'+'舒筋龙'+'刚压根'+'大活血'+'血藤'+'号筒草'+'红贯脚'+'酒虎杖'+'大绀著'+'太虫杖'+'甘除根'+'酸桶草'+'酸筒草'+'花斑竹'+'刚连根'+'叶蛇总管')

R=文摘

**Wan Fang database: From inception to 31^st^ July 2021**

主题: ('慢性阻塞性肺病'+'慢性阻塞性肺病急性加重'+'慢性阻塞性肺病急性发作'+'呼吸道感染'+'鼻炎'+'鼻窦炎'+'咽炎'+'喉炎'+'鼻咽炎'+'扁桃体炎'+'支气管炎'+'气管炎'+'咳嗽'+'感冒'+'外感'+'流感'+'喉咙痛'+'咽痛'+'咽喉痛'+'咽痒'+'鼻塞'+'流涕'+'咳'+'嗽'+'喘'+'肺'+'鼻'+'咽'+'喉') * 主题: ('虎杖'+'苦杖'+'斑杖'+'酸桶笋'+'斑根'+'酸汤杆'+'紫金龙'+'活血龙'+'阴阳莲'+'大叶蛇总管'+'九根龙'+'黄地榆'+'大虫杖'+'斑草'+'山茄子'+'搬倒甑'+'斑庄'+'斑庄根'+'武杖'+'鸟不踏'+'酸干'+'斑龙紫'+'野黄连'+'活血丹'+'舒筋龙'+'刚压根'+'大活血'+'血藤'+'号筒草'+'红贯脚'+'酒虎杖'+'大绀著'+'太虫杖'+'甘除根'+'酸桶草'+'酸筒草'+'花斑竹'+'刚连根'+'叶蛇总管')

主题= subject heading

**Sino-Med Database: From inception to 31^st^ July 2021**

1. ((("慢性阻塞性肺病"[常用字段:智能]) OR "慢性阻塞性肺病急性加重"[常用字段:智能]) OR "慢性阻塞性肺病急性发作"[常用字段:智能]
2. ((("呼吸道感染"[常用字段:智能]) OR "鼻炎"[常用字段:智能]) OR "鼻窦炎"[常用字段:智能]) OR "咽炎"[常用字段:智能]
3. ((("喉炎"[常用字段:智能]) OR "鼻咽炎"[常用字段:智能]) OR "扁桃体炎"[常用字段:智能]) OR "支气管炎"[常用字段:智能]
4. (("气管炎"[常用字段:智能]) OR "咳嗽"[常用字段:智能]) OR "感冒"[常用字段:智能]
5. ((("外感"[常用字段:智能]) OR "流感"[常用字段:智能]) OR "喉咙痛"[常用字段:智能]) OR "咽痛"[常用字段:智能]
6. ((("咽喉痛"[常用字段:智能]) OR "咽痒"[常用字段:智能]) OR "鼻塞"[常用字段:智能]) OR "流涕"[常用字段:智能]
7. (((((("咳"[常用字段:智能]) OR "嗽"[常用字段:智能]) OR "喘"[常用字段:智能]) OR "肺"[常用字段:智能]) OR "鼻"[常用字段:智能]) OR "咽"[常用字段:智能]) OR "喉"[常用字段:智能]
8. (#6) OR (#5) OR (#4) OR (#3) OR (#2) OR (#1)
9. ((((((("虎杖"[常用字段:智能]) OR "苦杖"[常用字段:智能]) OR "斑杖"[常用字段:智能]) OR "酸桶笋"[常用字段:智能]) OR "斑根"[常用字段:智能]) OR "酸汤杆"[常用字段:智能]) OR "紫金龙"[常用字段:智能] OR "活血龙"[常用字段:智能] OR "阴阳莲"[常用字段:智能] OR "大叶蛇总管"[常用字段:智能] OR "九根龙"[常用字段:智能] OR "黄地榆"[常用字段:智能] OR "大虫杖"[常用字段:智能] OR "斑草"[常用字段:智能] OR "山茄子"[常用字段:智能] OR "搬倒甑"[常用字段:智能] OR "斑庄"[常用字段:智能] OR "斑庄根"[常用字段:智能] OR "武杖"[常用字段:智能] OR "鸟不踏"[常用字段:智能] OR "酸干"[常用字段:智能] OR "斑龙紫"[常用字段:智能] OR "野黄连"[常用字段:智能] OR "活血丹"[常用字段:智能] OR "舒筋龙"[常用字段:智能] OR "刚压根"[常用字段:智能] OR "大活血"[常用字段:智能] OR "血藤"[常用字段:智能] OR "号筒草"[常用字段:智能] OR "红贯脚"[常用字段:智能] OR "酒虎杖"[常用字段:智能] OR "大绀著"[常用字段:智能] OR "太虫杖"[常用字段:智能] OR "甘除根"[常用字段:智能] OR "酸桶草"[常用字段:智能] OR "酸筒草"[常用字段:智能] OR "花斑竹"[常用字段:智能] OR "刚连根"[常用字段:智能]) OR "叶蛇总管"[常用字段:智能]
10. (#9) AND (#8)
